# Supplementary material for: Palm oil: Understanding barriers to sustainable consumption
Source: PLoS One. 2021 Aug 18;16(8):e0254897. doi: 10.1371/journal.pone.0254897 (PMC8372893; doi:10.1371/journal.pone.0254897)
Supplement: S1 File — (DOCX) [file pone.0254897.s001.docx]

**Complete Palm Oil-COM-B Survey**

There are many reasons why people do or do not purchase “green” or “sustainable” products that are **not harmful to the environment or do not deplete natural resources**. Please read each statement and indicate the extent to which it describes you.

1 = Not at all like me 2 3 4 5 = Just like me

I have heard about sustainable palm oil.

I know the difference between sustainable palm oil and ordinary palm oil.

I know which products contain palm oil.

I am aware of the effects of palm oil production on forests.

I am aware of the effects of palm oil production on certain animal species.

I am aware of how palm oil production impacts locals in Southeast Asia (farmers, plantation workers, etc.).

I can recognize brands which use sustainable palm oil in products.

Information about environmental impacts on product labels is often hard to understand.

I find green products expensive.

I am unwilling to pay more for green products.

I am often rushed for time when I go shopping for food and household supplies.

Getting the shopping done quickly is my top priority.

It is exhausting to change my purchasing behaviour for environmental reasons.

It is inconvenient for me to purchase green products.

Purchasing sustainably produced products is low in my list of priorities.

I regularly think about sustainability when shopping.

Despite my good intentions, I often forget to purchase green products.

I know where (e.g. in which stores) I can find sustainable products.

In a supermarket, I know where exactly (e.g. in which aisle) I can find green products.

I can easily find sustainably produced items where I usually shop.

The information given on product labels is insufficient to know if an item has been produced sustainably.

The information given on product labels is too small for me to easily read.

I rarely discuss environmental issues with my family and/or friends.

I rarely discuss shopping for green products with my family and/or friends.

I am worried that people will judge me for not buying green products.

Hardly any of my family and friends purchase green products.

My family and would be supportive if I purchased products with sustainable palm oil.

I feel a strong personal responsibility to buy green products.

It is important for me to purchase sustainable products.

Most of my purchases are habitual - that is, I tend to buy products that I have bought in the past.

It is hard to give up products I like, even though I know they are not good for the environment.

I rarely read all the ingredients on labels of products while shopping.

I am concerned about preserving our planet for future generations

I worry about the state of the planet, what we will leave behind for my children, grandchildren, and great grandchildren

I believe that consuming green products benefits my health.

I feel satisfied when/if I buy sustainable products.

I feel proud when/if I buy green products.

I find it very difficult to identify products that are sustainably produced.

I can influence others around me by encouraging them to shop sustainably.

I can make a difference to the environment if I purchase sustainably.

As a consumer, I have no power to bring about important environmental changes.

I carefully plan my purchases in advance so that I can buy green products.

I think that green products are often poorer in quality.

I think that sustainable products are less tasty.

I feel that green products offer poor value for money.

It is worth paying a higher price for green products.

I trust the information provided on labels about sustainability.

I feel guilty when I purchase products that are bad for the environment.

I see myself as a person who cares about the environment.

I see myself as a person who cares about animal life.

I empathize with animals that are affected by human farming activity.

I feel responsible for protecting the environment by purchasing green products.

I generally take animal welfare into account while shopping.
