# Supplementary material for: Palm oil: Understanding barriers to sustainable consumption
Source: PLoS One. 2021 Aug 18;16(8):e0254897. doi: 10.1371/journal.pone.0254897 (PMC8372893; doi:10.1371/journal.pone.0254897)
Supplement: Attachment — Submitted filename: Response to Editorial Team.docx [file pone.0254897.s002.docx]

**Response to Editorial Team**

**Thank you for reviewing the manuscript and pointing out issues to be addressed. Please see below the changes that have been made in the revised manuscript.**

One requirement is that the questionnaire or scale must be openly available under a license no more restrictive than CC BY. Accordingly, please include a copy of your questionnaire, scale, and/or interview guide as a Supporting Information file or provide a link if it is available through an online repository.

**Thank you for pointing this out. The entire scale is now available as S1. Appendix (pages 36 – 39), at the end of the manuscript. The items that were used in the analyses are presented in Table 2 (pages 18 – 19).**

Please also provide additional detail regarding how data extraction and analysis was performed on the qualitative component of your study, i.e., the interviews, including who conducted the interviews.

**Thank you for checking on this. Additional detail on who conducted the interview has been added (line 200, page 9). Detailed information on data extraction and analysis is written in another manuscript that has been submitted for publication. This has been mentioned as well (lines 211 – 212, page 10).**

Authors should include in the revised manuscript all the information removed for blind review, including the name of the institution(s) where the research was conducted.

**Thank you for pointing this out. The name of the institution has been mentioned in both places (line 192, page 9; line 355, page 16).**

We note you have mentioned a preliminary study was conducted where 13 Australian consumers were interviewed. Please provide additional information regarding participant consent for this preliminary study. Please specify if the consent was informed and if written or verbal consent was obtained. If verbal consent was obtained, please specify how it was documented. If the need for consent was waived by the IRB/ethics committee please let us know. Please add this information to both the ethics statement in your submission form and the ethics statement in your manuscript's methods.

**Thank you for checking. All participants provided written informed consent. This has now been included (line 199, page 9).**

We note in the ethics statement in your submission form you have provided the following information:

"The study was approved by the Human Research Ethics Committee of the University of New England (Approval No. HE19-223) and was conducted in compliance with the recommended research ethics procedure. All participants read an Information Sheet, after which they could provide implied consent by proceeding to the online survey."

Thank you for including this information.

Please also add this information to the ethics statement in your manuscript. Specifically, the ethics statement in your manuscript should include all information regarding participant consent and the full name of the ethics committee which approved the current study.

**Thank you for raising this. We had noted manner of participant consent under ‘Procedure’. However, as per the suggestion, we have shifted this information under ‘Ethics’ (lines 356 – 328, page 16).**
