# Supplementary material for: Palm oil: Understanding barriers to sustainable consumption
Source: PLoS One. 2021 Aug 18;16(8):e0254897. doi: 10.1371/journal.pone.0254897 (PMC8372893; doi:10.1371/journal.pone.0254897)
Supplement: Attachment — Submitted filename: Response to Editor & Reviewers_June 2021_Final.docx [file pone.0254897.s003.docx]

Response to Editor & Reviewers

# Response to Editor

**Please, restructure and report the 'preliminary study' as 'Study 1.' Doing so, sub-headings will also be needed to refer to the method and results of Study 1.**

*Thank you for this suggestion. This has been restructured as Study 1, as requested, detailing the sub-headings of participants, procedure, data analysis, ethics, and results. Please note that as mentioned in line 226-227 (page 10), this research study is under review in a more detailed format:*

Sundaraja, C.S., Hine, D.W., Alex, A., Cosh, S.M., & Lykins, A. (2021). Can consumers do it all? An exploration of factors that influence the purchase of sustainable palm oil products. *Manuscript submitted for publication (Journal of Food Products Marketing).*

**In addition to the scree plot, please run a parallel analysis**

*Thank you for this suggestion. We have additionally run a parallel analysis and described the same in the manuscript (lines 388-390, page18).*

**Was the dimensionality of the new measure items examined in additional two studies (and based on two different samples) to confirm the factorial validity and assess the predictive or nomological validity?**

*Thank you for this question. The factor structure was replicated in a subsequent study with a different sample.*

Sundaraja, C. S., Hine, D. W., Thorsteinsson, E. B., & Lykins, A. (2021). Purchasing products with sustainable palm oil: Designing and evaluating an online intervention for Australian consumers. *Manuscript submitted for publication (Journal of Environmental Psychology).* https://hdl.handle.net/1959.11/30061

*This information has now been included in the manuscript (lines 395-397, page 18).*

# Response to Reviewer #1

**Thank you for writing out most of the abbreviations in the text. I still think that using the abbreviation "COM-B" in the abstract is confusing, because many people won't know what it means and abstracts should really be crystal clear to the lay reader. COM-B is an element of BCW and not well known outside specific behavioural change studies.**
*Thank you for this comment. The acronym COM-B is expanded upon in lines 23-24 of the abstract.*

“The current study is the first to employ a capability-opportunity-motivation (COM-B) framework in green consumerism, to determine which capability, opportunity, and motivation factors strongly predict the intentional purchasing of sustainable palm oil products by Australian consumers (N = 781).”

**By the way, there are still no page or line numbers which makes it more difficult to comment on the text. There are some minor grammatical and spelling issues and I suggest that the authors proofread their text carefully before final submission.**

*Page numbers and line numbers are included. The manuscript has been proof-read once again – thank you.*  **Line 2 in Abstract: I suggest just writing: "expansion of oil palm plantations has led to the loss of tropical forests and biodiversity loss. Fire just isn't the biggest factor in deforestation.**

*Thank you – this line had been changed in the revised manuscript that was submitted, with the reference to fire removed (lines 22-23):*

“However, the growing demand for palm oil is leading to deforestation and biodiversity loss.”

# Response to Reviewer #2

**I would very much welcome it if the use of palm oil were briefly explained in more detail. Please point out that it is not only used in food and animal feed but also, for example, in cosmetics, detergents and also as fuel.**

*Thank you for this suggestion. We have included lines 71-77 including information on the use of palm oil.*

**The explanation on the preliminary study is good, but should already be written where the preliminary study is mentioned for the first time.**

*Thank you for this suggestion. This entire section has been re-written as Study 1, on the request of the editor.*

**I suggest that section “Participants section” and “Procedure” be placed directly one after the other.**

*Thank you for this suggestion. However, we believe that the current order of “Participants”, “Measures” and then “Procedure” is suited to describe this research study, as the measures used and described under “Measures” are subsequently referred to under “Procedure”. Changing this order might result in losing the coherent flow between these sections.*

**Table 3: Please explain all abbreviations. What is B, UB, LB etc.? Most of the readers will prob-ably know it but not everyone.**

*Thank you for this suggestion. We have now included a note under the table, expanding on all the abbreviations used (lines 425-429, page 21).*

**The discussion is very short and the comparison with the current literature is very poor. Please include more recent literature. A lot has been published on the topic of consumer preferences and palm oil in the last few years.**

*Thank you for pointing this out. The discussion has now been expanded upon and updated with more recent literature (References 91, 95, 97, 100, 101).*
